# CROPSR: an automated platform for complex genome-wide CRISPR gRNA design and validation

**DOI:** 10.1186/s12859-022-04593-2

**Published:** 2022-02-16

**Authors:** Hans Müller Paul, Dave D. Istanto, Jacob Heldenbrand, Matthew E. Hudson

**Affiliations:** 1 Illinois Informatics Institute, University of Illinois at Urbana-Champaign, Urbana, IL 61801 USA; 2 Center for Advanced Bioenergy and Bioproducts Innovation, Carl R. Woese Institute for Genomic Biology, University of Illinois at Urbana-Champaign, Urbana, IL 61801 USA; 3 Department of Crop Sciences, University of Illinois at Urbana-Champaign, Urbana, IL 61801 USA; 4 National Center for Supercomputer Applications, University of Illinois at Urbana-Champaign, Urbana, IL 61801 USA; 5 Present Address: OmniTier Inc., Milpitas, CA USA; 6 Present Address: ClosedLoop.ai, Round Rock, TX USA

**Keywords:** CRISPR, gRNA design, Bioinformatics pipelines, Soybean, Miscanthus, Crops

## Abstract

**Background:**

CRISPR/Cas9 technology has become an important tool to generate targeted, highly specific genome mutations. The technology has great potential for crop improvement, as crop genomes are tailored to optimize specific traits over generations of breeding. Many crops have highly complex and polyploid genomes, particularly those used for bioenergy or bioproducts. The majority of tools currently available for designing and evaluating gRNAs for CRISPR experiments were developed based on mammalian genomes that do not share the characteristics or design criteria for crop genomes.

**Results:**

We have developed an open source tool for genome-wide design and evaluation of gRNA sequences for CRISPR experiments, CROPSR. The genome-wide approach provides a significant decrease in the time required to design a CRISPR experiment, including validation through PCR, at the expense of an overhead compute time required once per genome, at the first run. To better cater to the needs of crop geneticists, restrictions imposed by other packages on design and evaluation of gRNA sequences were lifted. A new machine learning model was developed to provide scores while avoiding situations in which the currently available tools sometimes failed to provide guides for repetitive, A/T-rich genomic regions. We show that our gRNA scoring model provides a significant increase in prediction accuracy over existing tools, even in non-crop genomes.

**Conclusions:**

CROPSR provides the scientific community with new methods and a new workflow for performing CRISPR/Cas9 knockout experiments. CROPSR reduces the challenges of working in crops, and helps speed gRNA sequence design, evaluation and validation. We hope that the new software will accelerate discovery and reduce the number of failed experiments.

## Background

Over the past decade, the CRISPR bacterial system harnessed from *Streptococcus pyogenes* [1–3] has been optimized and become a revolutionary technique for genome editing, leading to the addition of alternative CRISPR systems to the toolbox [1, 4, 5]. One of the main challenges of this technology, since its inception, has been optimizing its cutting efficiency and specificity. Many bioinformatics tools have been developed to aid in this endeavour [6–10], with one of the most popular tools being developed by Doench et al. [11, 12]. One of the limitations of the current CRISPR design tools is that, for the most part, they revolve around the algorithm proposed by Doench et al.. The Doench method is currently considered the gold standard for CRISPR guide evaluation. This algorithm was designed based on mammalian genomes, and thus that task is where its performance is best. Many crops, however, exhibit paleopolyploidy as a result of genome duplication events. These events are evolutionarily favorable as they help increase genome diversity and the redundant alleles can undergo neofunctionalization under low selection pressure, improving adaptability to new environment or stress conditions [13]. Additionally, crop genomes are particularly affected by these duplication events, as specific, desirable traits, often associated with increased yield or stress resistance, have been positively selected over generations of breeding. RNA guide sequence evaluation models designed around mammalian genomes often do not address multiple gene copies properly, or at all, as these patterns are not frequently observed in these genomes. As a consequence, the scoring efficiency of such models for use on genomes of crop plants can be limited.

**Fig. 1:**
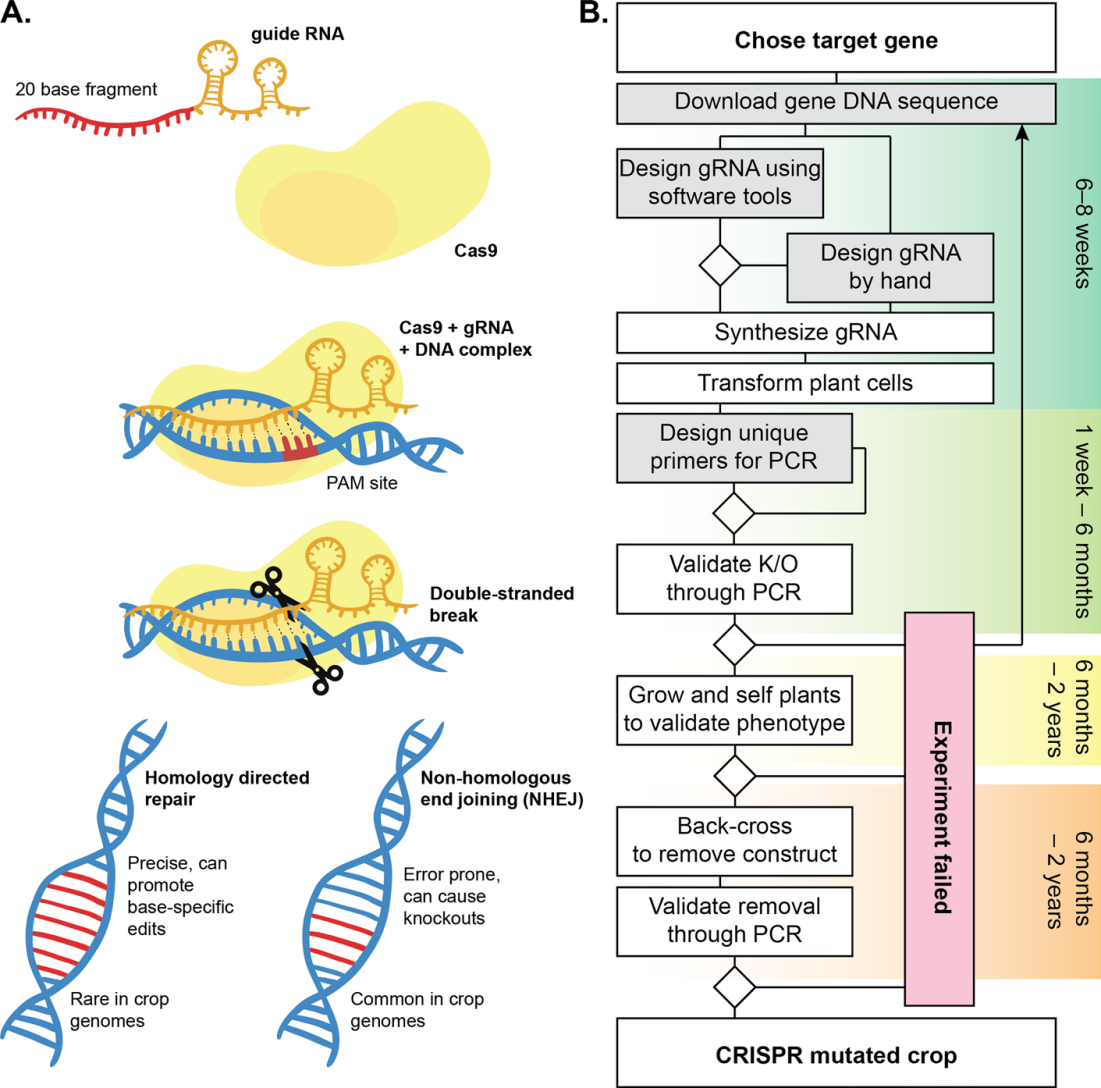
Overview of a typical CRISPR/Cas9 gene editing experiment. **A** Overview of CRISPR/Cas9 mechanism used to create deletions in crop genomes. **B** Diagram of a typical knockout editing experiment in a crop plant, with associated timeline. Improvements in the steps contained in gray blocks are anticipated from the CROPSR software

The CRISPR system, as a genome editing tool, is comprised of a protein component, a CRISPR-associated protein (Cas) and a single RNA molecule. The RNA has a structural domain that interfaces with the Cas protein, and a domain called the guide RNA (gRNA) that can be designed to compliment the DNA region that will be targeted. The Cas protein identifies the DNA based on a Protospacer Adjacent Motif (PAM), and once this location is identified the DNA double helix is unwound and the gRNA is bound to it. The Cas protein then promotes a double-stranded break in the double helix of the DNA before detaching from it. The cell’s native DNA repair systems can then either successfully repair the sequence by recombination, or fail, introducing mismatches or causing deletions, which is a common outcome in plant systems (Fig. 1A). One of the main advantages to this system is that to target a different portion of the DNA, there is only a small part of the system that needs to be redesigned: the 20 base long fragment of the gRNA that complexes with the target DNA. Designing the optimal sequence for the guide RNA is important in ensuring that the experiment will be more likely to grant the expected outcome: a successful mutation. This is increasingly important as the complexity of the target genome increases. In crops in particular, a complete CRISPR/Cas9 mutation experiment may take upwards of two years (Fig. 1B). Inadequately designed guides may take weeks, or potentially months, to reveal a failed attempt at targeted mutation. This interval is more valuable for season-sensitive crops, in which case the window for growing the plants may be limited and, for a failed experiment to be repeated, there could be a delay until the next growing season.

Here we present CROPSR, an open-source, automated platform that efficiently incorporates genome-wide gRNA design and evaluation, as well as allowing design of unique primers to facilitate experimental validation of the CRISPR/Cas9 knockouts through PCR, to increase the accuracy, efficiency and accessibility of CRISPR/Cas9 mutation experiment design. CROPSR has been specifically developed to be straightforward install and use on typical UNIX/HPC hardware, to be easily updatable to include new scoring models or Cas protein PAM sites, and to match or exceed the performance of current CRISPR/Cas9 gRNA design tools, particularly when used used on soybean, *Miscanthus* or other complex, repetitive and polyploid crop genomes. The newly developed model for evaluating gRNA sequences provided a substantial increase in quantity and quality of guide sequences provided compared to currently available tools, particularly for bioenergy crops such as the recently published *Miscanthus sinensis* genome [14].

## Methods

### Overview and required dependencies

CROPSR is a self-contained package written in Python 3, and it will not run on legacy versions (e.g. Python 2.7). Almost all necessary libraries are included with Python 3 as part of the standard library, with the two exceptions being Numpy (https://numpy.org) and Pandas (https://pandas.pydata.org). Both are widely used, well documented, and can be downloaded from the Python Package Index and installed using the ”pip” package installer. Note that for the analysis of larger genomes (2Gb or larger), Python 3.8 or newer is recommended to prevent an issue with a garbage collector bug present on 3.7 and under (this was not an issue with smaller genomes, tested at  750Mb).

### Input files and data pre-processing

To run genome-wide guide design, CROPSR requires the user to provide reference files for both the genomic DNA sequence from a FASTA file, and the genomic annotation from a GFF file. Since these files are user-provided they can be obtained from a variety of sources, including publicly available databases such as NCBI (https://www.ncbi.nlm.nih.gov) or Phytozome (https://phytozome.jgi.doe.gov), as well as user-generated data for novel unpublished genomes.

#### Genome sequence

Once the FASTA file containing the genomic sequence is imported, page breaks are removed and the text is formatted to account for differences in file formatting for FASTA files from different sources. The file is then converted into a Python dictionary, so sequences can be fetched by chromosome number.

#### Annotation files

For correlation of genomic features with the DNA sequence, CROPSR utilizes data from a GFF (preferably GFF3) file. Imported features, including genomic location, strand and phase, source of the GFF file, quality scores, and functional annotation are stored in a Pandas dataframe for ease of access later on. Although this is sufficient information to utilize genomes obtained from the NCBI, data originated from the Phytozome databases utilize a different structure and, thus, require an additional file.

*Handling annotation on Phytozome genomes* Due to Phytozome’s internal database organization, the GFF files provided are not associated with functional annotation. Rather, each entry is linked to a transcript name, which is then used to fetch functional annotation from a separate text file on the Phytozome website GUI. Hence, to link functional annotation to genomic locations, the “annotation_info.txt” for a specific genome is also required. This file can be found at the same folder as the GFF file when downloading from the database. When CROPSR identifies the source of the GFF as being Phytozome, it will look for the “annotation_info.txt” in the same folder, and with the same file name structure (e.g. a GFF file named “*file_name*.gff3” would cause CROPRS to attempt to open a file named “*file_name*.annotation_info.txt” from the same folder). Data from this secondary file is then appended to the function annotation field on the dataframe.

### Genome-wide gRNA design

One of the main factors that set CROPSR apart from other gRNA design software that are widely utilized is its capability to generate guides genome-wide, as well as being able to do so on a per-gene basis. A *gene* flag will prompt the input to require only a FASTA file, whereas a *genome* flag will require both FASTA and GFF. Most of the procedure described below is the same in both scenarios, but differences will be highlighted as needed.

#### Identification of PAM sites and guide design

PAM motifs for the specified CRISPR system will be identified by regular expression across the sequence, both in the sense and anti-sense strands, excluding the final bases on both the 5′ and 3′ ends. For each motif identified this way, a 20 base pair guide RNA will be designed following the instruction set for that CRISPR system (e.g. for Cas9, the guide will comprise the 20 bp upstream of the PAM motif). Then, a longer version of the guide sequence including 5 bp flanking on both the 5′ and 3′ ends is generated for on-site score calculation.

#### Guide evaluation

*On-site score* CROPSR has two different scoring algorithms implemented to evaluate guide sequences. The first is a modified version of the Doench algorithm [11]. For each guide, the following procedure is applied: first, the sequence is converted into two one-hot matrices, one first-order and one second-order, as per Doench’s description. Then, the first order matrix and second order matrix are multiplied by weight matrices obtained from [11] to generate the first order score matrix *I* and second order score matrix *J*, respectively. Guides are then assigned a score *k* based on their GC content ($k=-0.2026259$ if GC content $<50\%$, and $k=-0.1665878$ if GC content is $\ge 50\%$). A final on-site score *f*(*s*) is then calculated as a logistic regression, using 0.59763615 for the intercept [11], as shown on Eq. (1).

1 $$\begin{aligned} f(s) = \left[ 1+\exp ^{ -(int+k+\sum I_{r,c} +\sum J_{r,c} )}\right] ^{-1} \end{aligned}$$

The second is an algorithm based on a linear support vector regressor (SVR), designed for CROPSR to remedy problems identified in the algorithm described by Doench et al. [11]. The initial procedure is retained: the sequence is converted into two one-hot matrices, one first-order and one second-order, as previously described. The two one-hot matrices are then converted into a single vector, which is fed into a linear support vector regressor as the feature set. The model was trained utilizing the same dataset used by Doench et al. [11], and the feature set was modeled to predict the gene % rank, a continuous variable, and generate a score, rather than defining a threshold for classification (thus allowing lower-scoring guides to be accessed by users if required).

*Note on off-site score* CROPSR does not compute off-site scores when designing guides. Instead, guides that align to multiple locations on the genome are tagged, so they can be identified in the database, and either filtered out or used to edit paralogous regions in parallel. Currently, guides are considered to align to multiple locations if they have an exact match for the 20 bases, with no mismatches allowed. Inclusion of mismatches will be added in a future version, as it requires some hardware optimization to be implemented without a considerable impact to performance.

#### Data and associated metadata storage

Metadata including start and end positions, chromosome number, CRISPR system information, guide sequence, on-site score and a randomly generated unique ID for each guide are stored on a Pandas dataframe for ease of access.

### Functional annotation

The cutsite position, determined based on the specific CRISPR system, is utilized to fetch functional annotation by cross-referencing with the dataframe containing data from the GFF file input. Each entry on the GFF dataframe has both a start and end position fields, as well as chromosome number. Each cutsite has its functional annotation field appended by every GFF entry containing the cutsite in its start-end interval.

### Primer design

For each guide, a pair of PCR primers is also designed for quick experimental verification. Primer design is conducted utilizing an in-house algorithm on the *prmrdsgn* module, rather than depending on available pre-existing tools such as Primer3 [15, 16]. The module can accept either FASTA files or a MongoDB entry as inputs, with predominantly the same procedures being conducted. This grants the user flexibility to utilize this module as a standalone primer design tool, or as part of the CROPSR suite. For each input, pools of possible forward and reverse primer sequences are generated using regular expression. Melting temperatures ($T_{m}$) for each candidate in the pools are determined through thermodynamics parameters, assuming hybridization as a two-state process [17, 18]. Under this assumption, we can describe the thermodynamic parameters for forming single-stranded ($primer_{SS}$) primers from double-stranded primer ($primer_{DS}$) (Eq. 2).

2 $$\begin{aligned} primer_{DS}\leftrightharpoons primer_{SS} + primer_{SS} \end{aligned}$$

The equilibrium constant for this reaction is given by $K = \frac{\left[ primer_{SS}\right] \left[ primer_{SS}\right] }{\left[ primer_{DS}\right] }$. Van’t Hoff’s equation defines the relation between free energy, $\Delta G$, and *K* as $\Delta G^\circ = -RT\ln {K}$, where *R* is the ideal gas constant, *T* is the reaction temperature, in kelvin. Thus, we have the derived Eq. (3). The melting temperature, $T_{m}$, is the point in which when half of the double-stranded has been dissociated, which means $\left[ primer_{DS}\right]$ is then equal to half of its initial concentration, and $\left[ primer_{DS}\right] = \left[ primer_{SS}\right]$. Plugging these values and isolating for $T_{m}$, we have a way to predict the melting temperature based on the Gibbs’ free energy (Eq. 4) [19, 20].

3 $$\begin{aligned} \Delta G^\circ = RT\ln {\frac{\left[ primer_{SS}\right] \left[ primer_{SS}\right] }{\left[ primer_{DS}\right] }} \end{aligned}$$

4 $$\begin{aligned} T_{m} = \frac{\Delta G^\circ }{RT\ln {\frac{2\times \left[ primer_{SS}\right] }{\left[ primer_{DS}\right] }}} = \frac{\Delta G^\circ }{RT\ln {2}} \end{aligned}$$

Interactions between bases on different strands are influenced by neighboring bases, which is why the free energy at $37~^\circ {\text {C}}$, $\Delta G^\circ _{37}$, is calculated based on the nearest-neighbor model, as proposed by SantaLucia [21] and shown in Eq. (5). $\Delta G^\circ _{37} (init)$ is the sum of the free energies for bases on the extremities, and each $\Delta G^\circ _{37} (i)$ is one of the ten possible adjacent-base combinations, as listed on Table 1 [21].

5 $$\begin{aligned} \Delta G^\circ _{37} (pred) = \Delta G^\circ _{37} (init) + \sum _{i=1}^{10}n_{i}\Delta G^\circ _{37} (i) \end{aligned}$$

**Table 1:** Nearest-neighbor parameters for DNA/DNA duplexes

Nearest-neighbor sequence	$\Delta G^\circ _{37}$
$(5^{\prime } \rightarrow 3^{\prime }{/} 3^{\prime }\rightarrow 5^{\prime })$	(kJ × mol^−1^
AA/TT	−4.26
AT/TA	−3.67
AC/TG	−6.09
AG/TC	−5.40
TA/AT	−2.50
TC/AG	−5.51
TG/AC	−6.12
CG/GC	−9.07
GC/CG	−9.36
CC/GG	−7.66
Terminal A/T	4.31
Terminal C/G	4.05

Primers from the forward and reverse pools are then matched based on their melting temperatures to form primer pairs that work under the same PCR conditions.

#### Selecting unique primer pairs

For each primer pair, the amplified fragment length and sequence are recorded. The amplified sequence is aligned against the genome using bowtie [], to identify whether this pair generates a unique amplicon of the desired length. If multiple fragments appear with the same length and similar sequences, the primers are tagged so they can be verified for homeologous copies of the cloned fragment. Whenever possible, a primer pair without the homeologous tag will be selected and added to the dataframe for a specific guide RNA.

#### Using as a standalone tool

When using the *prmrdsgn* module of CROPSR as a standalone tool, a FASTA file for the reference genome is required. The procedure will be the same, except the alignment will be performed against the provided reference genome, and the primers will be provided as an output .CSV file instead of added to a dataframe.

### Output files

CROPSR outputs to a CSV file by default, but an option to output a Mongo database instead is provided.

## Results

**Fig. 2:**
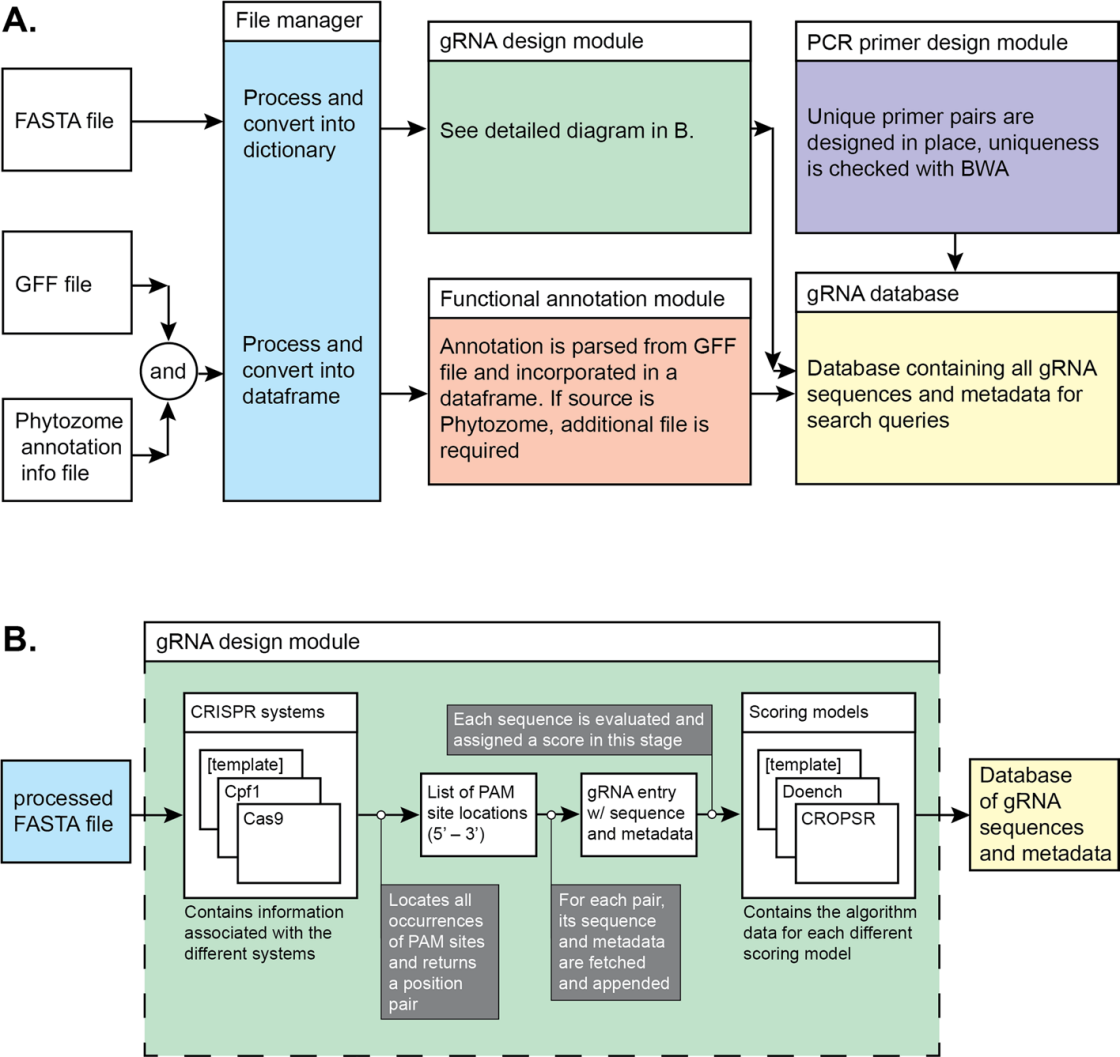
Functional block diagram of CROPSR modules. **A** The different input data files (FASTA, GFF, Phytozome annotation file) are imported and processed by multiple modular programs within the CROPSR suite. The genome sequence is submitted to the gRNA design program (shown in detail in **B**), and the output is placed in a MongoDB database (or optionally a CSV file). The GFF file, and Phytozome annotation file when applicable, are processed by a separate program, and then each entry in the database is updated with functional annotation to be used for search queries. Unique primer pairs are designed for each gRNA database entry. **B** The gRNA module takes data from the file manager module (which parses a FASTA input sequence file), and generates a list of location pairs ($5^{\prime }$–$3^{\prime }$) for every PAM site match. The sequence, strand, start and end positions and CRISPR system for each guide are stored, and a score representing expected performance of each potential gRNA is calculated utilizing one of the available algorithms. Final data for each guide is then added to the database to be associated with functional annotation and PCR primers for validation

We have developed CROPSR, a tool to design, evaluate, and validate CRISPR sgRNA in biofuel crop genomes. The code for CROPSR was written in Python 3.7, and is available at https://github.com/cabbi-bio/cropsr. To the best of our knowledge, CROPSR is the first tool developed for genome-wide generation and validation of CRISPR sgRNA in crop genomes. One of the main advantages of CROPSR is that it was developed as a single, modular, self-contained tool that performs all steps in the experimental design—from identifying the PAM sites located in the genome, to the design of PCR primers for experimental validation (Fig. 2).

### Feature comparison

Although there are tools available that provide a similar set of functions for gene-based analysis and that allow the task to be performed in batches [6], they are comprised of a pipeline of preexisting methods and, thus, have limitations similar to those of the packages they include. Additionally, batch-based analysis is limited by a gene-based design, which does not necessarily consider applications for regulatory, non-coding regions.

CROPSR was designed to be a self-contained tool, requiring minimal dependencies to function. Its modular nature brings more control and flexibility to the user. Most parameters can be set via arguments in the command line, which is desirable for supercomputer applications.

#### Primer design

Existing tools, such as Primer3 [15, 16], are widely adopted for designing PCR primers for bacterial, fungal, and mammalian genomes. For this reason they are often included in pipeline-based genomic tools for CRISPR sgRNA design, such as Chopchop [6–8]. Due to the polyploid nature of many crop genomes, however, primers need to be designed in a way that allows independent validation of mutations in different gene copies. Implementations of primer design on currently available tools either do not validate whether the amplicon has matches elsewhere in the genome, or perform a simple alignment and discard primer pairs that have multiple hits. The first scenario may cause primers that target more than one location in a polyploid genome, whereas the second may cause the tool to be unable to provide primers for a specific region. We have included a PCR primer design module within CROPSR’s algorithm in an attempt to help mitigate these issues by providing information on additional gene copies that may be affected by the same guide, while also potentially identifying and validating guides that may affect only a single copy of a duplicated gene, when possible.

Additionally, CROPSR’s primer design module calculates the melting temperatures ($T_{m}$) based on the nearest-neighbor model proposed by SantaLucia [21]. This method provides more accurate temperatures for longer sequences when compared to Bolton and McCarthy [22], used in Primer3. Additionally, the nearest-neighbor method adopted provides better consistency in regions with varying GC contents, which is often an issue in repetitive, A/T-rich crop genomes.

#### Revised model for sgRNA scoring

There are two well-established tools that are most commonly used for designing sgRNA for CRISPR knockout experiments in plants, each with its advantages. Chopchop [6–8] provides a pipeline that facilitates guide sequence design and calculates a score that is predictive of the guide’s on-target efficiency, as well as its off-target potential. This score is based on the methods proposed by Doench et al. [11, 12], which utilized a support vector machine classifier to segregate the designed guides in two groups: the top $20\%$ performing, classified as optimal, from the bottom $80\%$. Additionally, the method penalizes guides that can target the genome in more than a single location, as this characteristic can promote undesirable, non-specific edits. One of the shortcomings of this method, in crops specifically, is that many crops have paleopolyploid genomes. In such cases, most essential genes will have multiple copies throughout the genome, due to the presence of multiple copies of the gene, and/or whole-genome duplication events in the evolutionary history of that crop. Thus, in crop genetics applications, the elimination of guides that have A/T content outside a hard-coded range, or that hit in more than one location, may result in the software being unable to output any guides for some genes. Additionally, for some experiments it may be desirable to target all of the paralogs in a particular group [23].

CRISPR-P [9, 10] is a web-based tool developed for designing sgRNA for CRISPR systems in plants. Its algorithm [24] calculates a score based on the sgRNA’s chance to hit off-target positions. A score is then calculated for a guide based on the number of matches it displays throughout the genome, including a limited number of mismatches. This approach helps mitigate the effect of discarding guides that may hit at many locations, improving the user's capability to design guides for crop genes that exhibit multiple copies. One of the shortcomings of this method, however, is that it does not calculate a score for on-site binding, as adopted by other tools like Chopchop.

Although the aforementioned models have important qualities and can correctly design functional guides for many applications, both present limitations for a number of potential CRISPR/Cas9 editing applications in crop systems, for example those employed by Dong et al. [23]. Hence, to circumvent these limitations, we developed a model based on a slight variation of the algorithm proposed by Doench et al. [11] and adopted by Chopchop. For our model, we utilized the training data set provided by Doench et al., that was utilized to generate the original model they describe, and followed their methodology. Through this process we identified parameters that may be interfering with the guide design for A/T-rich crop genomes, such as melting temperatures at distinct portions of the 20 base sequence and a GC-content threshold, and removed these parameters from the input of our modified model. We then compared a number of alternative scoring algorithms to the one employed by Doench et al. The data was fed into four different types of supervised learning models, a support vector machine (SVM) classifier as used by Doench et al., a linear support vector regressor (SVR), a random forest regressor (RFR), and a multi-layer perceptron regressor. Using leave-one-group-out as the cross-validation method, we evaluated and compared each scoring algorithm. Based on these results, we opted for the SVR scoring algorithm to incorporate into CROPSR, as it performed best with the available data while retaining many of the desirable characteristics of the original method.

Additionally, we do not use the off-site scoring model used in other approaches (Chopchop uses Doench et al. [12], CRISPR-P uses Hsu et al. [24]) where the off-site hit generates a large penalty in the score of the guide. As this penalizes the score directly and prevents the design of guides that target multiple genes, it prevents a key use of CRISR/Cas9 in crop systems. We have addressed the presence of potential off-site hits in our output file by adding a tag and instructing the user to check for potential additional copies of the targeted region. This is based on an assumption that designing a guide that is capable of editing all copies of a given gene, instead of a single copy, is a likely goal when generating gene knockouts in crop genomes. Separating the off-site hits from the scoring algorithm allows us to provide the user with the power to decide whether a guide that hits the genome at more than one locus is desirable or undesirable.

### Benchmarking

We compared the guide sequences designed by CROPSR, Chopchop, and CRISPR-P for four syntaxin genes from soybean, previously characterized by our group using manually designed gRNAs after existing software failed to design guides [23]. These genes were problematic because they require guides in A/T-rich regions of a crop genome, and have highly similar sequences to one another. In addition, for this project it was necessary to target both each gene individually, and all of the genes simultaneously. CROPSR outperformed the other software tools for every gene in either total number of guides designed, number of guides designed with a score $\ge 0.8$, or in both (Table 2). The choice of score cutoff was based on the threshold defined in the algorithm by [11], utilized by Chopchop [6–8], to discard under-performing guides. The only gene for which CROPSR did not suggest the largest total number of guides was Syn16, where CRISPR-P suggested one guide that CROPSR did not. However, CROPSR outperformed CRISPR-P in genes with scores above 0.8 by the same amount. For the other three genes, CROPSR and CRISPR-P suggested the total number of guides, however CROPSR’s scoring algorithm awarded a larger number of guides with scores above 0.8. Chopchop designed fewer guides for all four genes compared to the other two tools, and was outperformed by CROPSR in number of guides with scores above 0.8 for all genes.

**Table 2:** Number of guide sequences generated by CROPSR and other currently available software for four syntaxin genes from soybean

Soybean	Number of guides with score $\ge 0.8$ (total number of guides)		
Syntaxin			
Gene	CROPSR	Chopchop	CRISPR-P
Syn02	2 (141)	0 (137)	0 (141)
Syn12	9 (217)	3 (196)	2 (207)
Syn13	13 (396)	2 (386)	2 (396)
Syn16	6 (386)	2 (378)	5 (387)

#### Improvements to the sgRNA scoring model

**Fig. 3:**
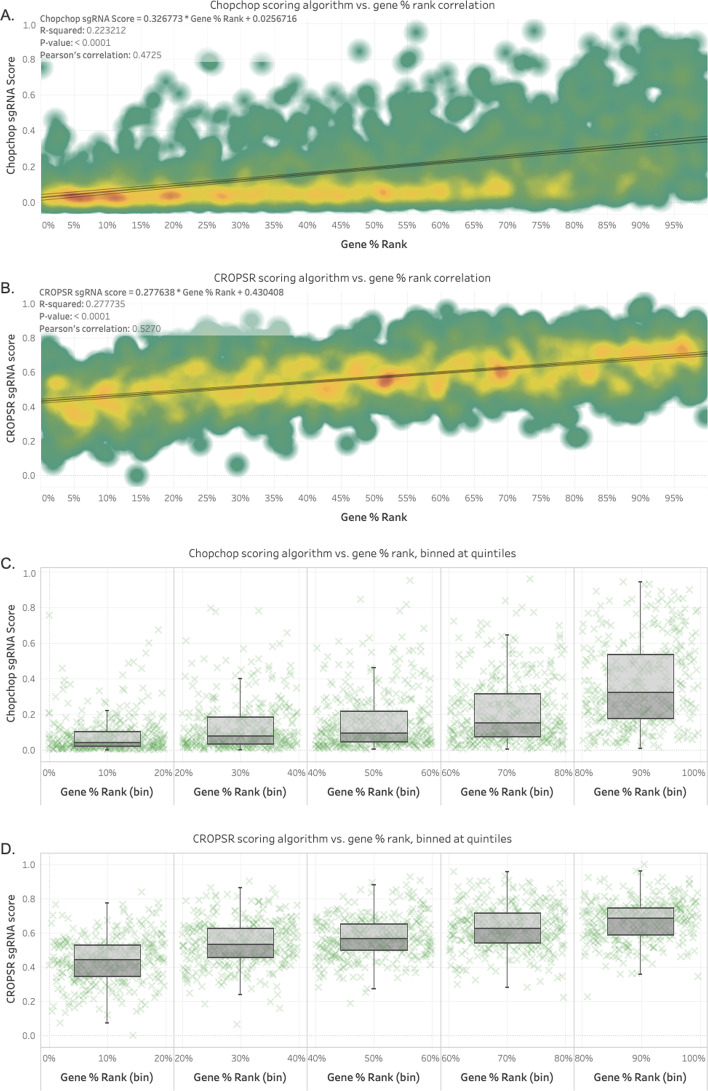
Comparison of the scoring performance of CROPSR with the Chopchop algorithm. **A** Density plot of the score generated by the Chopchop scoring algorithm against the “gene % rank”, a ranking of experimentally-determined relative performance of gRNAs on a per-gene basis. **B** Density plot of the CROPSR scoring algorithm against the gene % rank. **C** Binned scatter + box plot of the Chopchop scoring algorithm against the gene % rank. The gRNA targeted for experimental use by Chopchop are those in the 80–100% bin. **D** Binned scatter + box plot of the CROPSR scoring algorithm against the gene % rank

The adoption of a regression-based model for the prediction of the gene % rank for sgRNAs provided a reduction of approximately 19% in the variance explained by the model, as represented by the $r^2$ increase from 0.223 for the model adopted by Chopchop (Fig. 3A) to 0.278 obtained for CROPSR (Fig. 3B). Likewise, the Pearson’s correlation between the independent variable, gene % rank, and the dependable variables (the scores), was improved by $11.5\%$ for CROPSR compared to Chopchop. Another benefit of the model implemented in CROPSR is that the adoption of a quantitative approach improved the correlation with rank across the entire range, but the effect is noticeably larger in the high-scoring ($\ge 0.8$) gRNAs compared to the model implemented by Chopchop (Fig. 3C for Chopchop, Fig. 3D for CROPSR). Additionally, a comparison of the variances within the target bin (80–100%, the highest-performing quintile) utilizing the Kruskal-Wallis method confirmed that the scores generated by Chopchop and CROPSR for that particular group are not part of the same distribution (*p*-value = 0.02788).

#### Whole-genome evaluation

One of the goals when developing CROPSR was to provide users with the ability to design guide sequences targeting regulatory regions of crop genomes. Regulatory or non-coding regions often exhibit repetitive sequences and their genetic structure can widely vary compared to coding, gene regions. These traits can cause currently available software tools to have difficulty designing guide sequences in non-coding regions. Although differences in parameters such as melting temperatures and G/C content have an effect when evaluating on-target activity of a guide RNA sequence, the presence of repetitive sequences in non-coding regions has a bigger impact on the prediction of off-target activity. Due to the repetitive nature of the DNA in regulatory regions, it is more likely that guide RNA sequences originated in such regions will be matched to multiple different locations in the genome. This can cause the scoring algorithms used in these software tools to heavily penalize guide sequences designed to target non-coding regions, which may lead to no guide sequences being considered viable by such programs.

The approach adopted in CROPSR to circumvent these issues is to take the whole genome as an input rather than the sequence of a single gene or region. By splitting the genome by chromosome, it is possible to identify all potential target locations by scanning for the PAM sites, and then designing the guide sequences for each site following any requirements of the specific Cas system (e.g. for Cas9, the sequence is designed as the 20 bases located upstream of the PAM site). Start and end positions for each guide, as well as the cut-site for the Cas nuclease activity and a calculated on-site score are then added as metadata to each guide sequence.

Each guide sequence is aligned against the entire genome to identify potential sequences that target more than a single location, however no penalty is applied to the score in positive cases. A tag is added to sequences that hit in multiple locations, and the user may then decide whether these guides are of interest (e.g. for mutating all copies of a gene) or not. This results in a much larger number of guide sequences being provided compared to the currently available alternatives, and is especially useful for the complex genomes of energy crops. A whole-genome analysis of a complex genome, such as *Miscanthus sinensis* [14], can generate upwards of 200 million potential CRISPR targets (Table 3).

**Table 3:** Number of gRNA sequences generated by CROPSR for *Miscanthus sinensis*

Chromosome in	# of guide sequences
*Miscanthus sinensis*	Generated by CROPSR
Chr01	15,174,305
Chr02	14,634,737
Chr03	10,933,532
Chr04	11,515,578
Chr05	11,539,743
Chr06	11,947,192
Chr07	16,147,361
Chr08	9,662,343
Chr09	8,046,957
Chr10	7,389,629
Chr11	8,198,766
Chr12	8,558,113
Chr13	6,745,249
Chr14	5,959,044
Chr15	7,181,726
Chr16	8,132,384
Chr17	8,480,072
Chr18	8,443,163
Chr19	8,851,088
Unplaced scaffolds	19,158,456
Total	206,699,438

### Run time, memory usage and disk space

The full analysis of the genome of *Miscanthus sinensis* using CROPSR required a minimum of 16 Gb of RAM, and a quad-core processor. Memory usage peaked at around 12 Gb. The total run time under these specs was 6 days, 7 h, 17 min and 40 s. The .*csv* file output is 26.94 Gb in size.

## Discussion

As previously described, CROPSR is a tool developed from the ground up as an open source Python application to perform all steps required to design guide and primer sequences for genome editing, with additional consideration paid to the complications of performing CRISPR/Cas9 editing in complex, often polyploid crop genomes, such as the need to target multiple paralogs and the need for unique validation primers.

### CROPSR use cases

The development of CROPSR was inspired by the limitations of current gRNA sequence design tools for A/T-rich regions of crop genomes. However, we quickly realized that the polyploid genomes often found in crops also impose limitations to algorithms that score based on guide sequence uniqueness. This often causes otherwise useful gRNA sequences that target multiple homeologous copies of a gene to be filtered and not accessible to the user. Finally, unique validation primers for each target site are often the limiting factor in polyploid genomes or for high-copy genes, thus the design of these is integrated into CROPSR. Although CROPSR was developed with polyploid, A/T-rich crop genomes in mind, it is the first tool for genome-wide gRNA sequence design that we are aware of, and can be employed in any genome. Additionally, due to its modular nature, modules can be utilized individually to design PCR primers for a given sequence, or to originate gRNA sequences for a single gene rather than a whole genome.

We have purposefully designed CROPSR as a set of modular tools to facilitate the implementation of new functionality, such as new scoring algorithms as they become available, or new CRISPR systems as needed. An additional advantage of this approach is that individual modules can be utilized separately from the complete package. For example, the PCR primer design tool can be used as a stand-alone application for any PCR experiment, or the CRISPR guide design tool can be used for an isolated gene sequence (for example, for an in vitro, controlled experiment where matches elsewhere in the genome are not a consideration).

### Improved scoring model

When developing the new scoring model for CROPSR, we gave special consideration to avoiding imposing penalties on guide sequences that match at more than a single location on the target genome. Crop genomes can possess multiple copies of important genes, and penalizing guides that match these genes at more than a single locus often causes guide sequences designed to target these genes to have poor scores. Such penalties against repeated sequences are implemented in Chopchop and many other tools [11, 12, 24]. These scoring algorithms have been developed to facilitate CRISPR experiments in humans that target single genes. In plant biology, it is sometimes desirable to target multiple paralogous sequences simultaneously, and in the case of many genes in some genomes, this is the only option. Therefore, removing guides that target multiple sequences by default before reporting results becomes an obstacle when attempting to design guide RNA sequences for crop genomes. As existing optimizations frequently discard guide sequences with a low score at an early stage, and scores are often heavily penalized for targeting multiple locations, such software tools can be unable to provide the user with gRNA sequences for certain plant genes. The approach adopted in the model utilized by CROPSR does not apply penalties for sequences with multiple hits even when scoring is performed utilizing the implemented version of the Doench [11] algorithm. However, sequences with multiple hits can easily be filtered from the reported results.

To further improve the reliability of the provided gRNA sequences, a new algorithm based on a linear support vector regression method was employed. This choice was based primarily on the nature of the data being analyzed. The methodology adopted by Doench et al. [11], which inspired our approach, initially converts nucleotide sequences into one-hot matrices. A weight matrix can then be obtained from a population of one-hot matrices, based on the correlation between base frequency at specific positions (a continuous variable) and an effect state. This is where both methods diverge, however. Doench et al. set a threshold at an ordered rank data setting to create two discrete classes, which then were used to train a classifier to segregate the highest efficiency sequences from the remaining population. The threshold was defined to maximize the odds of a successful CRISPR experiment (in human and mouse genomes), and simplifies the choice for the user. We have instead opted for a different approach, attempting to predict where in the ordered rank a new data point would be situated. We opted for assisting the user to make an informed decision about whether a guide is suited for an experiment or not, based on a combination of the ranking score (provided by CROPSR) and field-specific information.

Another important factor in repetitive genomes is the ability to design primers to validate edits. Thus, although gRNAs that target multiple genes can be desirable, it is necessary to be able to design unique PCR primers to amplify each target site and sequence the edited region. For this reason, primer design is integrated into the CROPSR software suite, and can be used to filter CROPSR-designed gRNAs for those allowing the design of unique primer sets.

### New workflow for CRISPR experiments

**Fig. 4:**
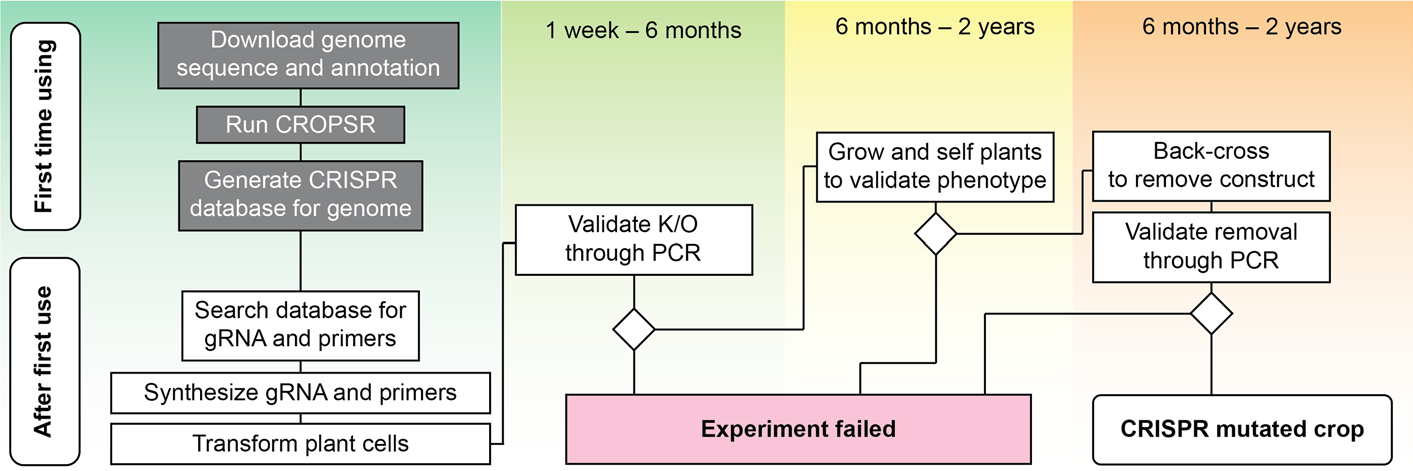
Overview of a CRISPR experiment using CROPSR Timeline and steps of a typical CRISPR/Cas9 knockout experiment in a crop plant genome, utilizing CROPSR. Steps contained in gray blocks represent steps that only need to be done once per genome, at the first utilization of CROPSR (database generation). Consecutive uses on the same genome require only a database search, as shown

With CROPSR, the workflow for CRISPR experiments in crop genomes can be revised and simplified (Fig. 4). During the first utilization of CROPSR with a specific genome, a full analysis and genome-wide guide sequence design will be performed. All gRNA sequences and their important metadata will be stored in a database for ease of identification. This step, although time-consuming, only needs to be performed once per genome. On subsequent uses, a search to the database will return all data required to perform the experiment: the gRNA sequence, cut site location and genome annotations matching that position, a pair of unique PCR primers for experimental validation and the melting temperatures (*Tm*s) for the primer pair. Grouping all the steps on a single platform provides ease of use and helps minimize variations, either from different experiments using various different tools, or even between different operators utilizing the same pipeline.

### CROPSR limitations, in comparison to other tools

To utilize CROPSR’s features fully, a whole-genome run is recommended prior to performing the CRISPR experiments. While this run only needs to be done once for each organism, and the results can then be stored in a database, the initial run requires significant server-class compute resources. The time required to perform this process should be kept in mind when planning experiments utilizing this tool. This step is required on the first use for a given genome, to generate a database containing all potential gRNA sequences and their associated metadata. On subsequent utilization, database searches should provide the user with the necessary guide and primer sequences with minimum further calculation, and can be delivered through a web interface.

### Future directions

The release of CROPSR is the first step on a longer path to provide an innovative, open source toolkit to assist in the design of CRISPR experiments and other manipulations of crop genomes. During development and testing of CROPSR, the challenges that emerged have helped develop improved strategies for future work.

#### Additional output formats

One of the consequences of the genome-wide approach adopted in CROPSR is the size of the output file. Processing large, complex genomes result in a colossal number of guide sequences with associated metadata, such as functional annotation and primers for PCR validation. In an effort to make the output file accessible to the majority of users, the options currently provided are CSV and and MongoDB. Future releases may include support for other database formats, targeted at optimizing storage size and search functionality without compromising the ability to update the stored data with new information.

#### Novel scoring algorithms

The current implementation allows the utilization of both the model developed by Doench et al. [11], as well as the one developed for this work. Functionality aimed at facilitating the addition of new models will be a part of future releases. Additionally, novel scoring algorithms based on different sets of features to positional frequency of nucleotides will be explored in an attempt to provide better information to the user and further facilitate the design of CRISPR experiments.

#### Hardware scaling optimization

The server-class compute time of a genome-wide analysis, such as the one performed by CROPSR, is very significant. Due to the modular, open source nature of CROPSR, new parameters and models that get added in the future will cause the current compute times to get increased further. To mitigate some of this effect, as well as help minimize issues associated with the long compute times, future plans include a revision of the CROPSR code to provide better hardware scaling optimization. In our opinion the benefits to be gained from having the tool available immediately outweigh those of waiting for non-essential optimization updates.

## Conclusions

We have developed CROPSR, an open source tool for genome-wide design and evaluation of gRNA sequences for CRISPR. In an effort to provide the scientific community with a tool aimed at facilitating the design of genomics experiments by minimizing the out-of-lab time, CROPSR is capable of creating complete, searchable databases containing genome-wide information needed for CRISPR experiments, including unique primer pairs for validation through PCR. The improved scoring model adopted by CROPSR represents a significant improvement over currently widely utilized methods. Additionally, CROPSR provides the user with all information required to decide whether the generated gRNAs are fit for an experiment instead of deciding for them. This change will greatly benefit crop scientists, as previously available scoring models could be unreliable for complex crop genomes. CROPSR allows data output as CSV and MongoDB, with more formats being planned for future addition, together with optimizations to reduce compute time and novel scoring algorithms.

## Data Availability

The CROPSR package, tutorial and sample data are hosted at https://github.com/cabbi-bio/cropsr.A Docker container version is also available at https://hub.docker.com/r/h2muller/cropsr.

## Abbreviations

CRISPR: Clustered regularly interspaced short palindromic repeats
Cas9: CRISPR associated nuclease
DNA: Deoxyribonucleic acid
RNA: Ribonucleic acid
SVM: Support vector machine
SVR: Support vector regressor
PCR: Polymerase chain reaction
CSV: Comma-separated values
